# Emergence of dengue virus serotype 2 in Mauritania and molecular characterization of its circulation in West Africa

**DOI:** 10.1371/journal.pntd.0009829

**Published:** 2021-10-25

**Authors:** Toscane Fourié, Ahmed El Bara, Audrey Dubot-Pérès, Gilda Grard, Sébastien Briolant, Leonardo K. Basco, Mohamed Ouldabdallahi Moukah, Isabelle Leparc-Goffart

**Affiliations:** 1 Unité des Virus Emergents (UVE), Aix Marseille Université - IRD 190—INSERM 1207, Marseille, France; 2 Institut de Recherche Biomédicale des Armées, Marseille, France; 3 Institut National de Recherche en Santé Publique, Nouakchott, Mauritania; 4 Aix Marseille Univ, IRD, AP-HM, SSA, VITROME, Marseille, France; 5 IHU—Méditerranée Infection, Marseille, France; 6 Unité de Recherche Génomes et Milieux, Faculté des Sciences et Techniques, Université de Nouakchott Al-Aasriya, Nouakchott, Mauritania; 7 Initiative mauritanienne pour la lutte contre les maladies endémiques “MEDCINGO” Nouakchott, Mauritania; Oregon Health and Science University, UNITED STATES

## Abstract

The number of sporadic and epidemic dengue fever cases have reportedly been increasing in recent years in some West African countries, such as Senegal and Mali. The first epidemic of laboratory-confirmed dengue occurred in Nouakchott, the capital city of Mauritania situated in the Saharan desert, in 2014. On-site diagnosis of dengue fever was established using a rapid diagnostic test for dengue. In parallel, the presence of *Aedes aegypti* mosquitoes in the city was confirmed. The initial diagnosis was confirmed by RT-PCR, which showed that all samples from the 2014 dengue epidemic in Nouakchott were dengue virus serotype 2 (DENV-2). The whole genome or envelope protein gene of these strains, together with other DENV-2 strains obtained from travelers returning from West African countries to France between 2016 and 2019 (including two Mauritanian strains in 2017 and 2018), were sequenced. Phylogenetic analysis suggested a recent emergence of an epidemic strain from the cosmopolitan genotype belonging to West African cosmopolitan lineage II, which is genetically distinct from African sylvatic genotype. The origin of this DENV-2 lineage is still unknown, but our data seem to suggest a recent and rapid dispersion of the epidemic strain throughout the region. More complete genome sequences of West African DENV-2 are required for a better understanding of the dynamics of its circulation. Arboviral surveillance and outbreak forecasting are urgently needed in West Africa.

## Author summary

In Africa, dengue viruses 1 to 4 are transmitted to primates by *Aedes* mosquitoes in a sylvatic cycle or an urban/epidemic cycle involving humans. Infection in humans may be asymptomatic or may range from mild flu-like illness to severe hemorrhagic fever. The dengue viruses have a pantropical distribution, mostly in urbanized areas where vectors have become well adapted. This report shows dengue can also emerge in desert areas, as seen for the first time in an outbreak in 2014 in Nouakchott, Mauritania, where climatic, environmental, and human behavioral changes favor the emergence of *Aedes* mosquitoes. Dengue virus 2 was found in multiple human samples collected during the outbreak. Genomic analysis of dengue virus 2 isolates from the Mauritanian outbreak and from infected travelers revealed the rapid emergence of a specific “West African epidemic strain” of the virus throughout West Africa during the last decade, which is distinct from other strains found elsewhere and from historical or sylvatic strains. More genomic data would help us understand the circulation of dengue virus in West Africa, to help forecast and mitigate outbreaks in this region.

## Introduction

Dengue fever is a mosquito-borne disease present in many tropical and sub-tropical regions of the world and is one of the major global threats to public health [1]. Approximately 40–60% of the world’s population is living in areas at risk for dengue virus (DENV) infection [2]. Dengue viruses, which belong to the *Flaviviridae* family and *Flavivirus* genus, are transmitted to humans through the bites of *Aedes* spp. mosquitoes. DENV infection is usually asymptomatic or associated with mild, flu-like symptoms, but it may occasionally, i.e. in 5–10% of cases, develop into life-threatening hemorrhagic fever or shock syndrome. Dengue viruses present 4 distinct serotypes (DENV-1, DENV-2, DENV-3, and DENV-4). Infection by one serotype confers long lasting protective immunity for that serotype. Within each serotype, strains have been phylogenetically grouped into genotypes [3].

South and Central America and Asia are the most affected regions. Sporadic cases or epidemics of DENV have been reported from several African countries, most frequently in East Africa [4]. It was at one time thought during the second half of the 20^th^ century that yellow fever and dengue are mutually exclusive and that Africa, where yellow fever occurs, is relatively spared from dengue infection, while yellow fever is absent but dengue is common in Asia [5]. However, all four serotypes have been detected in Africa: DENV-1 and DENV-2 in Nigeria in 1964–1969 [6, 7], and 20 years later, DENV-3 in Mozambique and DENV-4 in Senegal [8–10]. Three major epidemics involving DENV-1 and DENV-2 occurred in Africa: in the Seychelles, 1976–1977 (DENV-2), in Djibouti, 1991–1992 (DENV-2), and in the Comoros islands, 1992–1993 (DENV-1) [8–10]. More recent epidemiological data have led to growing awareness of DENV circulation in Africa, and reports of DENV in Africa have been increasing as standardization and dissemination of molecular techniques have made identification of the etiologic agent of an epidemic more accurate [11].

Despite the progress made, the actual DENV burden is still not well known in West Africa [2, 12, 13]. Laboratory-confirmed cases of dengue and molecular studies on DENV circulating in the African continent are rare. In Mauritania, West Africa, no documented case of dengue fever had been reported prior to 2014. In neighboring countries, all 4 DENV serotypes have been identified in Senegal since 1970 [14], whereas DENV-1 and DENV-2 were detected in Mali in 2006 [15]. An entomological investigation conducted in Nouakchott, Mauritania’s capital, in mid-2014 revealed the establishment of *Ae*. *aegypti*, an urban anthropophilic mosquito and highly competent vector for DENV which has been regularly captured since then [16, 17]. This was followed by an epidemic of dengue fever in late 2014. In the absence of previous epidemiological data on DENV in the country, the aims of the present study were to determine the serotypes of DENVs collected in Nouakchott during the 2014 outbreak, to compare the sequences of these 2014 Mauritanian DENV strains with those of other DENV collected from travelers returning to France from Mauritania in 2017 and 2018, and to analyze the phylogenetic relationships of Mauritanian strains of DENVs with DENVs detected in travelers returning from other West African countries to France between 2016 and 2019.

## Methods

### Ethics statement

The study conducted in Mauritania was part of the research project approved by the *ad hoc* institutional ethics committee of Université de Nouakchott Al-Aasriya and the institutional ethics review board of the Institut de Recherche pour le Développement (Marseille, France). Enrolled adult patients provided verbal informed consent during consultation. Samples collected in France were residual sera of suspected cases of dengue fever addressed to the French National Reference Center for Arbovirus for molecular diagnosis. Patient’s consent to research is entailed to analysis request form provided by the patient’s clinician.

### Laboratory investigations of the outbreak in Nouakchott

Between September and November 2014, febrile adult patients consulting at Teyarett Health Center and Mother-Child Medical Center of Nouakchott were enrolled. A rapid diagnostic test (RDT) for malaria (SD Bioline Pf/Pan test; Alere, https://www.alere.com) was performed to exclude malaria as *Plasmodium vivax* and *Plasmodium falciparum* parasites are endemic in Nouakchott, and transmission peaks from September to November [18]. Patients with negative malarial RDT or a positive RDT for malaria with persistent symptoms despite effective antimalarial chemotherapy were tested for dengue with a RDT (CareStart dengue combo NS1 + IgM/IgG; Access Bio, Inc., Somerset, NJ).

A total of 27 patients with positive dengue RDT were enrolled in the study, two with coinfection dengue-malaria. After verbal informed consent, 5 ml of blood sample were collected by venipuncture, centrifuged (2,000 *g* for 10 min), and the plasma was stored at -20°C.

Plasma samples were thawed and imbibed onto Whatmann 3MM filter papers, air dried, and sent at room temperature to the French National Reference Center for Arbovirus for molecular testing in 2019. Viral RNA was extracted from dried plasma samples as described by Vongsouvath *et al*. [19] and tested for DENV-1 to 4 RNA by real-time RT-PCR [20].

### Imported dengue cases in France

In France, a network for the epidemiological surveillance and control of various communicable diseases is under the coordination of national reference centers. The surveillance of arboviral diseases of humans, including dengue fever, is ensured by the French National Reference Center for Arbovirus, which gathers data on autochthonous and imported cases of arboviral diseases, establishes laboratory diagnosis, and offers expertise in the field and medical advice to medical professionals. As part of its mission, the French National Reference Center for Arbovirus receives blood samples of suspected cases of dengue fever from various hospitals in France for molecular diagnosis.

Twelve DENV-2 PCR-confirmed blood samples from travelers returning to France from West African countries between 2016 and 2019 (including 1 from Mauritania in 2017 and another 1 from Mauritania in 2018) were included in the present study. Viral RNA was extracted from fresh sera or plasma using the EZ1 Advanced XL automat (Qiagen, Germany) and the EZ1 Virus mini kit, according to the manufacturer’s recommendations. Real-time RT-PCR was performed to detect the presence of DENV-2 RNA [20].

### Genomic analysis of DENV-2 strains acquired in West Africa

Full genome sequencing was attempted directly on PCR-positive Mauritanian dried plasma samples and on PCR-positive blood samples from travelers returning to France from West Africa between 2016 and 2019. All next generation sequencing (NGS) was performed at the Faculty of Medicine, Unit of Emerging viruses (Marseille, France), as described in an earlier study [21]. Briefly, 4 overlapping fragments covering the complete DENV-2 genome were amplified using SuperScript III RT/Platinum *Taq* high fidelity (Invitrogen, France). Equimolar solution of purified amplicons were then sequenced by next-generation sequencing using Ion Torrent Personal Genome Machine (ThermoFisher Scientific, Waltham, Massachusetts, USA) and consensus sequences were obtained using CLC Genomics Workbench software (V11.0.1, CLC Bio).

Sequencing of the envelope gene was performed using the same method developed by Baronti *et al*. [21] with specific primers shown in Table 1 to amplify 3 overlapping fragments.

**Table 1:** Primers targeting DENV-2 envelope gene sequence.

Target	Primer	Sequence	Size (bp)
D2.protE.S1	Den2_F819	5’-GRTCYTGAGACATCCAGGYTT-3’	720
	Den2_R1539	5’-TGCAGCARCACCATCTCATT-3’	
D2.protE.S2	Den2_F1235	5’-AGAGGATGGGGAAATGGAT-3’	814
	Den2_R2049	5’-GGYTCTGCTTCTATGTTGACT-3’	
D2.protE.S3	Den2_F1871	5’-GAAATAGCAGAAACRCAACATGGAA-3’	674
	Den2_R2545	5’-GARGGGGATTCTGGTTGGAA-3’	

Phylogenetic analysis of the complete genome of DENV-2 was performed with a dataset of all available complete sequences deposited in the GenBank (n = 356), including those of West African strains, and aligned against the reference sequences of DENV-1, DENV-2, DENV-3 and DENV-4 strains. DENV-2 complete coding sequence alignment used for the complete coding sequence phylogenetic analysis was completed with 382 complete envelope gene sequences available in GenBank selected on the basis of > 95% nucleotide identity or country of origin in Africa, including historical and sylvatic strains. Sequence alignment and phylogenetic analysis were performed using Molecular Evolutionary Genetics Analysis (MEGA) version 6.0 software [22]. Phylogenetic tree was constructed using maximum likelihood method based on the general time reversible (GTR) model and discrete γ distribution with evolutionarily invariant sites, as recommended by the nucleotide substitution model selection algorithm implemented in the software [23].

## Results

Real-time RT-PCR confirmed the presence of DENV-2 in 26 of 27 (96.3%) samples collected in Nouakchott in 2014 but failed to detect DENV RNA in one sample. Sequencing was attempted in all PCR-positive samples, i.e. 26 Mauritanian samples obtained in Nouakchott in 2014 and 12 West African (including 2 Mauritanian) samples from imported dengue cases in 2016–2019. Complete genome sequences were successfully obtained for 8 of 12 DENV-2 imported cases in France (Table 2). The dried plasma samples of Mauritanian strains from the 2014 outbreak did not allow the recovery of complete viral sequence. Complete envelope gene sequences were obtained from 7 of the 2014 Nouakchott samples, as well as the remaining 4 DENV-2 imported from Benin, Côte d’Ivoire, Mali, and Togo. All 19 sequences (8 complete genome sequences and 11 complete envelope gene sequences) were deposited in GenBank (Table 2).

**Table 2:** Characteristics of dengue virus strains analyzed in this study.

Isolate code	Country	Date	Nature of sample	Sequence obtained	GenBank accession no.
2014/MR/B843	Mauritania	Nov-2014	dried plasma	envelope gene	MT981512
2014/MR/B854	Mauritania	Nov-2014	dried plasma	envelope gene	MT981494
2014/MR/B860	Mauritania	Nov-2014	dried plasma	envelope gene	MT981492
2014/MR/B862	Mauritania	Nov-2014	dried plasma	envelope gene	MT981514
2014/MR/B864	Mauritania	Nov-2014	dried plasma	envelope gene	MT981513
2014/MR/B868	Mauritania	Nov-2014	dried plasma	envelope gene	MT981491
2014/MR/B870	Mauritania	Nov-2014	dried plasma	envelope gene	MT981511
2019/TG/CNR51609	Togo	30/06/2019	serum	envelope gene	MT982918
2019/SN/CNR50154	Senegal	04/01/2019	plasma	full genome	MT981148
2017/MR/CNR46460	Mauritania	30/11/2017	plasma	full genome	MT980927
2018/MR/CNR50012	Mauritania	13/12/2018	serum	full genome	MT981085
2018/SN/CNR49422	Senegal	15/10/2018	serum	full genome	MT981011
2016/BJ/CNR37671	Benin	19/05/2016	plasma	envelope gene	MT982919
2017/CI/CNR44006	Côte d’Ivoire	06/06/2017	serum	full genome	MT982169
2017/CI/CNR44064	Côte d’Ivoire	13/06/2017	serum	envelope gene	MT982922
2017/ML/CNR46292	Mali	08/11/2017	serum	envelope gene	MT982917
2016/BF/CNR41166	Burkina Faso	02/11/2016	serum	full genome	MT982731
2019/BF/CNR52121	Burkina Faso	10/08/2019	plasma	full genome	MT982126
2019/BF/CNR52631	Burkina Faso	08/09/2019	plasma	full genome	MT982148

**Fig 1:**
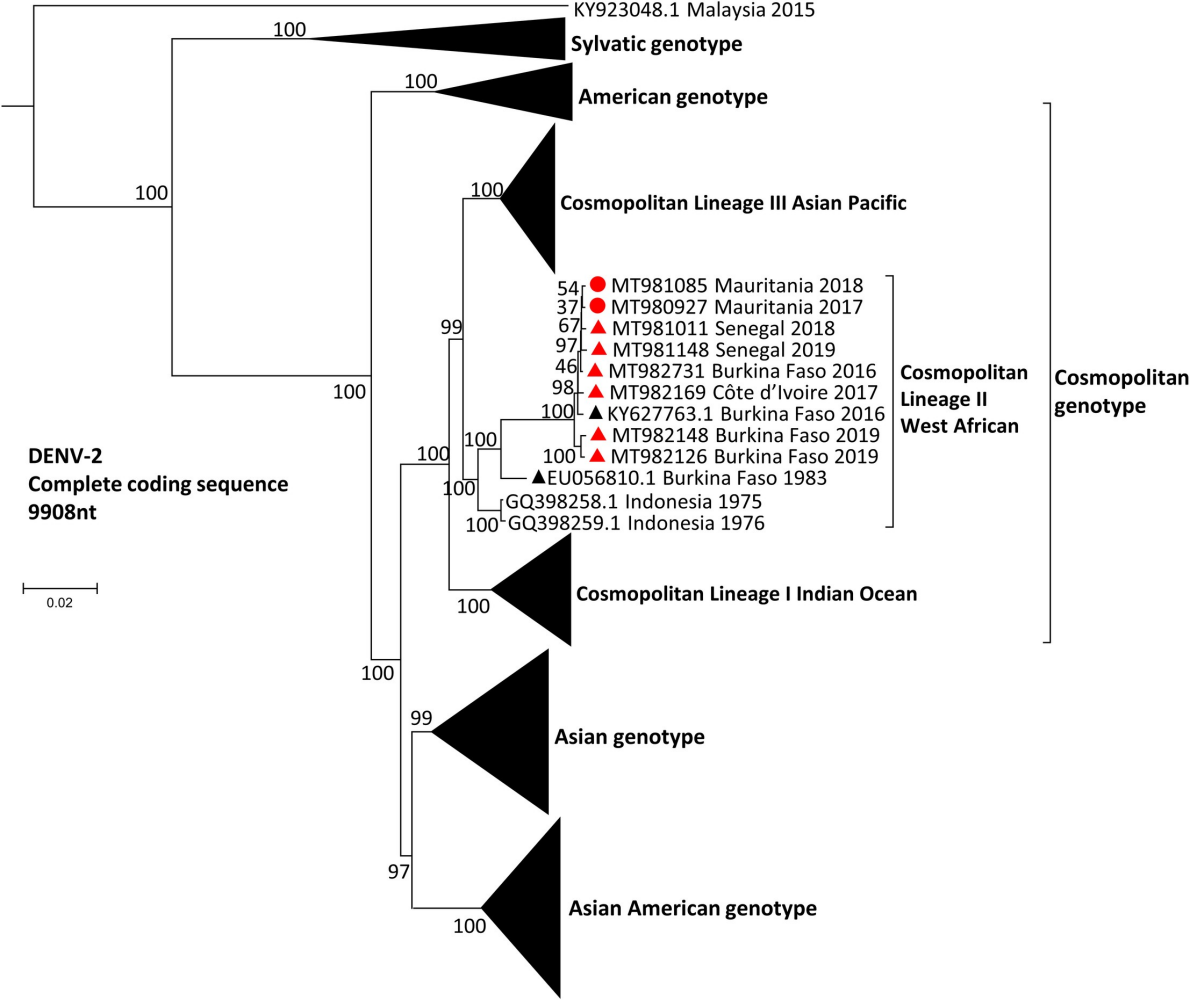
Maximum-likelihood phylogenetic tree based on complete genome of DENV-2 strains. DENV-2 strains from our data set are indicated by red circles for Mauritanian strains and red triangles for strains from other West African countries. Each strain is identified by its GenBank accession number, country of origin and year of sample collection. Reference DENV-2 genotyping alignment was completed with complete coding sequences available on GenBank selected based on nucleotide identity (>95%) or country of origin (Africa). Bootstrap support values (percentage of 1000 replicates) are shown at nodes. Scale bar indicates genetic distance (nucleotide substitutions per site). The tree was rooted with reference strains of DENV-1 (MH888331.1), DENV-3 (MH888333.1), and DENV-4 (MF004387.1) (not shown). Scale bar indicates nucleotide substitutions per site. DENV, dengue virus.

**Fig 2:**
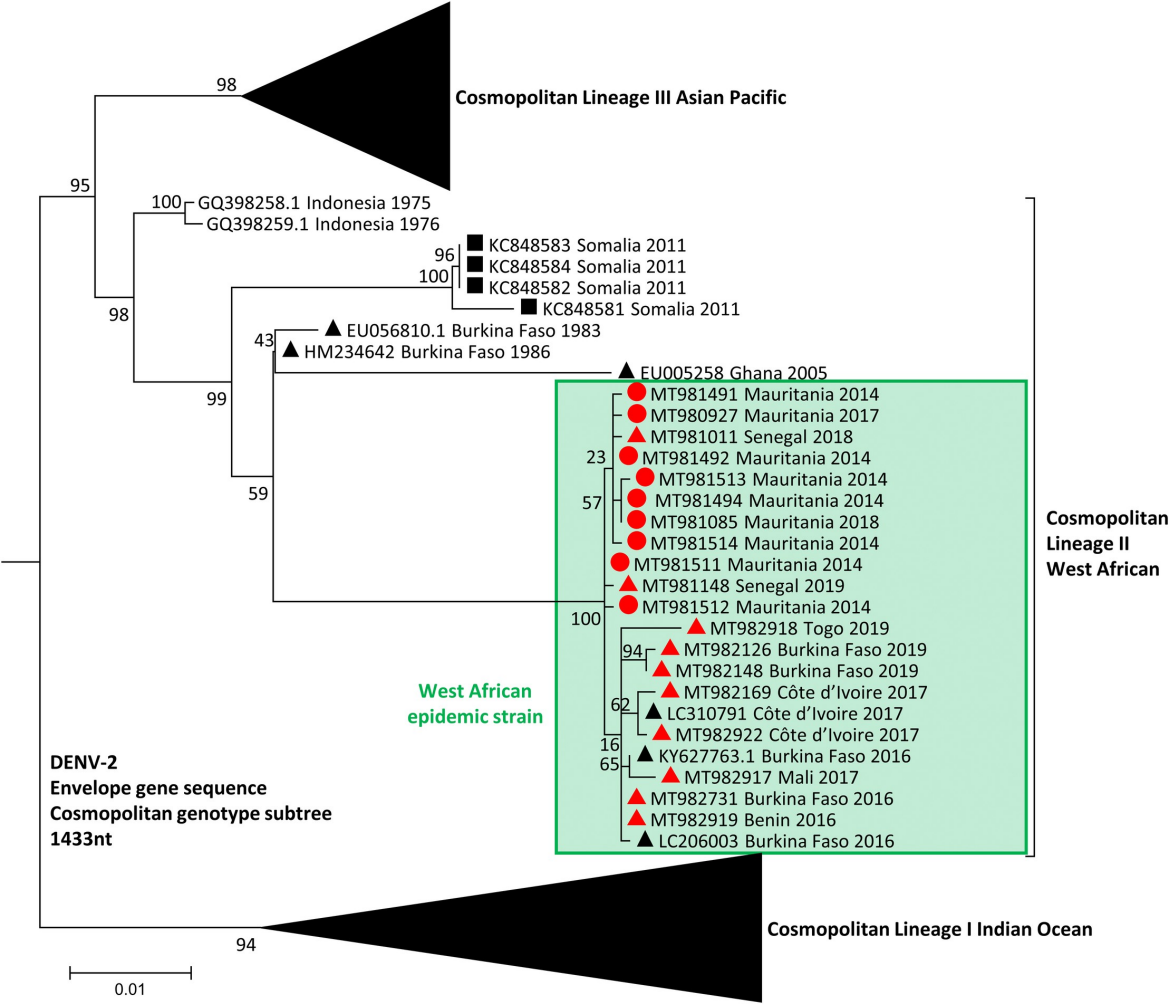
Maximum-likelihood phylogenetic tree based on envelope gene sequence of DENV-2 strains. DENV-2 strains from our data set are indicated by red circles for Mauritanian strains and red triangles for strains from other West African countries. Sequences retrieved from GenBank are indicated by black triangles for West Africa and black squares for the rest of Africa. The tree was constructed using 382 envelope gene sequences. DENV, dengue virus.

Phylogenetic analysis of complete coding sequences revealed that all West African isolates collected during the period between 2016 and 2019 grouped together with a 2016 Burkina Faso isolate (GenBank accession no. KY627763.1) (Fig 1), within the previously described lineage II of the cosmopolitan genotype [24]. Viral sequences in this group share over 99.4% nucleotide pairwise identity and 99.7% amino acid identity, suggesting a recent common ancestor for those 8 strains. Those strains differ from the historical strains circulating in West Africa (Senegal 1970, 1974 and 1999, Nigeria 1966, Burkina Faso 1980, Côte d’Ivoire 1980, Guinea 1981; GenBank accession no. EF105380-91, EF457904 and EU003591), which all belong to the sylvatic genotype as represented in Fig 1. Moreover, phylogenetic analysis of the envelope gene sequences showed that DENV-2 samples from the 2014 Nouakchott outbreak and the travel-acquired DENV-2 belong to the same viral group, supported by 100% bootstrap value (Fig 2). Viral sequences in this group share over 99.2% nucleotide pairwise identity and 97.9% amino acid identity in the E-gene, confirming that all 19 of our strains detected in West Africa have a recent common ancestor. All together, these data suggest that DENV-2 viruses encountered in West Africa since 2014 can be considered as members of the same viral strain, now referred as the “West African epidemic” strain (Fig 2).

## Discussion

Dengue virus is known to be transmitted through two relatively compartmentalized transmission cycles, with genetically distinct DENV genotypes: sylvatic and urban cycles. Sylvatic DENVs are maintained in a sylvatic enzootic cycle involving non-human primate reservoirs and arboreal mosquitoes (*Ae*. *luteocephalus*, *Ae*. *taylori*, *Ae*. *furcifer*, *Ae*. *opok* and *Ae*. *africanus*) [25]. Spillover infection of the sylvatic cycle may occur and can be responsible for the emergence of dengue fever in humans. Sylvatic DENV-2 is generally thought to be less pathogenic in humans and has been associated with at least one outbreak in Senegal in 1990 and one hemorrhagic dengue fever case [26, 27]. *Aedes aegypti*, which was initially zoophilic, originated from a sylvatic form in Africa and has adapted to urban environment, became anthropophilic through adaptation, and spread throughout tropical Africa and beyond as a domestic form [28, 29]. Both the descendant domestic mosquito (i.e., *Ae*. *aegypti aegypti*) and the sylvatic ancestral mosquito (i.e., *Ae*. *aegypti formosus*) are present in Africa. Epidemic DENVs are maintained in an urban cycle between humans and anthropophilic mosquitoes, such as *Ae*. *aegypti* and *Ae*. *albopictus*, which have adapted to domestic environments. African *Ae*. *aegypti* populations exhibit lower infection and dissemination rates than pan-tropical populations, which may partially explain the lower incidence of dengue in Africa, compared with Asia and Central and South America [30–32].

*Aedes* spp., including *Ae*. *aegypti*, have been known to be present in southern sahelian Mauritania since the late 1960s [17, 33, 34]. Some of the *Aedes* spp. found in southern Mauritania (e.g., *Ae*. *aegypti*, *Ae*. *vittatus*, *Ae*. *metallicus*, *Ae*. *luteocephalus*) can potentially transmit yellow fever virus, either experimentally or naturally, but symptomatic or fatal cases of yellow fever have rarely occurred in the country [33, 35]. Rift Valley fever, which is also transmitted by *Aedes* spp., notably by *Ae*. *vexans*, *Ae*. *sudanensis*, and *Ae*. *ochraceus*, has been reported in humans in recent years in Mauritania [17, 36, 37]. Despite the presence of several species of *Aedes*, mostly in southern Mauritania, dengue fever had never been suspected nor documented prior to 2014, most likely because malaria is known to be the predominant febrile disease in this zone and has often been treated on the basis of presumptive clinical diagnosis until the advent of affordable disease-specific RDTs in the early 2010s in Mauritania. It was only in 2014 that *Ae*. *aegypti aegypti* and *Ae*. *caspius* were captured for the first time in Nouakchott [16]. The capital city is situated in the Saharan zone of the country where it can be hypothesized that climatic and environmental conditions have been unfavorable for a long time for the establishment of *Ae*. *aegypti* larval habitats. In recent years, however, Nouakchott has undergone major urban transformation, including improved coverage of potable water supply from the Senegal River in 2010, but has been suffering from a lack of waste water evacuation system. In 2013, the amount of annual rainfall largely exceeded that of preceding and following years (130 mm in 2013 vs 63–66 mm in 2011–2012 and 82–87 mm in 2014–2015), resulting in extensive flooding of parts of the capital city for weeks in September 2013. After flooding, it has been observed elsewhere that the population of *Aedes* mosquitoes can explode due to simultaneous hatching of mosquito eggs laid in the soil [38, 39]. These adverse weather and environmental conditions could possibly have contributed to the occurrence of dengue epidemic in the following year, i.e. in 2014. Entomological surveys conducted in Nouakchott and other cities in the country have shown the presence of *Aedes* spp. after 2014 [17], which is in agreement with the reported occurrence of dengue fever in other Mauritanian cities as recently as in 2020.

Elsewhere in the countries that share a common border with Mauritania, the number of sporadic cases and dengue epidemics is reportedly increasing in recent years. In Senegal, with which Mauritania shares a 742 km border, *Ae*. *aegypti aegypti* is common and has been incriminated in recent dengue epidemics [40–42]. Dengue fever is endemic in Senegal [43–50]. During the last decade, all four DENV serotypes have been found in different regions of Senegal. In some epidemics, a co-circulation of two DENV serotypes was observed [44, 48]. In Mali, *Ae*. *aegypti* and *Ae*. *albopictus* are also present, including in Bamako, the capital city [51, 52]. An earlier study conducted in samples collected in 2006 suggested a co-circulation of yellow fever virus and DENV (serotypes 1 and 2) in the country [15]. In the 2010s, a major dengue epidemic has not been documented in the country, but a seroprevalence study on 376 blood samples collected in 2009–2013 from febrile, symptomatic patients without malaria or yellow fever showed DENV IgM and/or immunoglobulin G (IgG) by enzyme-linked immunosorbent assay (ELISA) (which detects all 4 serotypes), suggesting that dengue fever may be one of the important causes of febrile illness in the country [53]. A cluster of dengue fever (n = 16, including the index case) in a district in Bamako was confirmed by molecular diagnostic methods in 2017, but unserotyped [54]. Molecular data from our sample set place DENV-2 in Mali during the same year. In other countries to the north of Mauritania, i.e. Algeria, Morocco, and Western Sahara, there is no transmission of arboviruses by *Aedes* mosquitoes in humans, and only imported cases are occasionally seen [55–57].

Since its first isolation in Nigeria in 1966 up to 1980, DENV-2 strains isolated in West Africa belonged to the sylvatic genotype. The detection of the Burkina Faso 1983–86 strains (GenBank accession no. EU056810.1 and MH234652) clustering with Indonesia 1975–1976 strains (GenBank accession no. GQ398258.1 and GQ398259.1) marks the presence of non-sylvatic DENV-2 in West Africa. This lineage has remained undetected in Africa for 30 years until the isolation of 2 additional strains in Ouagadougou, the capital of Burkina Faso, in 2016 (GenBank accession no. KY627762.1 and KY627763.1) [58]. Continuous circulation of the 1983 strain in the country was hypothesized to be maintained locally [58]. Our phylogenetic analysis and high identity (> 98.8% amino acid identity; > 97.1% nucleotide identity) between the West African epidemic strain and the Burkina Faso 1983 strain tend to support this hypothesis. Phylogenetic analysis of DENV-2 strain characterized in 2017 in Louga city, Senegal, situated about 330 km from Nouakchott (the two cities are connected by a route via Saint Louis and Rosso), also showed that the Senegalese DENV-2 strain is closely related to strains from a dengue epidemic that occurred in Burkina Faso in 2016 (KY62776.1) [48, 58]. The genetic similarity of recent West African isolates may suggest a relatively recent and rapid geographical dispersion of the epidemic strain rather than a progressive dispersion undetected for 30 years. Additional strains would be required to determine the date and origin of that emergence and further consolidate this hypothesis. Major regional sporting events such as the ECOWAS Games, first set in Nigeria in 2010, then in Ghana in 2012, could be one of the contributing factors to such a rapid dispersion in West Africa.

Another molecular study on samples collected in Mauritania (the exact location was not specified) in 2014, as in our study, confirmed the presence of DENV in the country [59]. The authors of that study showed the presence of DENV-1 in two Mauritanian samples, as determined by serotype-specific real-time reverse transcriptase loop-mediated isothermal amplification (RT-LAMP). The results of those findings were not confirmed by sequencing. Since the 2014 dengue epidemic in Nouakchott, the Mauritanian Ministry of Health has reported other dengue fever epidemics as late as in 2020, not only in Nouakchott, but also in the northern cities of Atar and Zouérate [60, 61]. These reports are generally based on the local health information system and RDT for dengue after ruling out malaria and/or COVID-19 by other disease-specific RDTs and have not been confirmed by more sophisticated diagnostic procedures. Nonetheless, in addition to our study on imported cases of dengue in France in 2017 and 2018, other authors have confirmed the presence of DENV in Mauritania after the 2014 epidemic in Nouakchott. During an epidemic of Rift valley fever in Mauritania in 2015, blood samples collected from 184 patients with suspected hemorrhagic fever in 26 health centers located in different areas of the country were analyzed, and co-circulation of DENV and Rift valley fever virus was confirmed by RT-PCR in 8 patients [62]. In that study, 19 additional patients were RT-PCR-negative but positive for dengue IgM by ELISA.

Despite the scarcity of molecular data at present, there seems to be sufficient evidence supporting the emergence of DENV in Mauritania in 2014 and that epidemics occur regularly, probably throughout the country. The causes of the emergence of DENV in Mauritania have not been elucidated, but the presence of *Aedes aegypti* and other *Aedes* spp. is now established. Several hypotheses can be advanced to explain viral transmission. First, an increasing commercial exchange between West Africa and Asia favors a long-distance dispersal of pathogens and/or their vectors [63]. Several Asian countries have become principal trading partners of Mauritania, and business travel between Asia and Mauritania occurs on a daily basis. In Senegal and Mali, it has been suggested through molecular and phylogenetic analysis that DENV-1 outbreaks in 2015–2019 were due to a single introduction of the virus from Asia [47]. Moreover, used car tires, which are well-known habitats and means of dispersal of *Ae*. *aegypti* [64, 65], have been massively imported into the country from Asian countries where dengue is endemic. *Aedes aegypti* eggs, known for their resistance to desiccation, may have been inadvertently transported from Asia and brought into the country. However, our phylogenetic analysis does not support a recent Asian introduction of DENV-2 strains currently circulating in West Africa.

Second, mosquito vectors and/or viremic human hosts may have migrated or transported from neighboring countries, in particular from Senegal where dengue fever occurs frequently [43–49]. Dengue fever also occurs in Mali, but possibly to a much lesser extent [15, 53, 54].

Third, vertical (i.e. female adult to eggs) and sexual (i.e. adult male to adult female) transmissions of DENV in *Aedes* spp. have been reported [66]. This phenomenon implies that a viremic human host or non-human primate may not be required to initiate sporadic or epidemic transmission of DENV in a new site.

Fourth, a hypothesis of an incursion of the sylvatic cycle into urban areas cannot be ruled out. The dominant geographic feature of southern Mauritania and its neighboring countries to the south and east is sahelian. Historically, dengue epidemiology has been characterized by the circulation of sylvatic DENV in West Africa [67–69]. A classical sylvatic cycle of arboviruses maintained by non-human primates (principally patas monkeys, *Erythrocebus patas*) and forest-dwelling mosquito vectors has been demonstrated for DENV-2 in some West African countries (Burkina Faso, Guinea, Côte d’Ivoire, and Senegal), but it is not known if the sylvatic cycle of other DENV serotypes occurs in Africa [25, 69–71]. This classical epidemiological feature has undergone a major change, with *Ae*. *aegypti* adapting to urban environment and becoming the major vector for human to human transmission of the virus. Although Mauritania does not border any forest area in the region, the geographical features of the Sahel do not exclude possible sylvatic cycle maintained by *Aedes* spp. and the ground-dwelling *Erythrocebus patas* monkeys, which are the only extant non-human primates in southern Mauritania [72]. It has also been strongly suggested that patas monkeys can be naturally infected with DENV from human hosts [73]. This hypothesis would require the “West African epidemic strain” of DENV to be introduced and maintained in the local sylvatic cycle, as postulated in South America [74]. Based on the geographic distribution of *Aedes* spp. and mobility of *E*. *patas* monkeys in the sahelian West Africa, further investigations on the relationship between the existing sylvatic cycle and emerging urban transmission cycle are required to gain more insight into the rapidly changing DENV epidemiology in West Africa [75].

Molecular data derived from imported cases illustrate the importance of diagnosis and surveillance of travel-acquired infections. Indeed, previously available data from Benin, Togo and Mali are scarce, incomplete (unserotyped or probable cases), and/or based on indirect antibody testing of imported cases [15, 76–79]. Our analysis of imported cases has allowed to detect DENV-2 in Benin and Togo and to characterize it molecularly in Mali.

The present study has several limitations. The number of PCR-positive samples was relatively small despite the occurrence of a dengue epidemic in Nouakchott in 2014. Due to the unpreparedness of the country to anticipate dengue epidemic after the detection of *Aedes aegypti* in 2014 in Nouakchott and the fact that dengue epidemic occurred during the peak malaria transmission season (September to November) in the capital city [16], health authorities, medical personnel, and patients were mostly unaware of the origin of febrile illness once malaria was excluded. At that time, RDT for dengue was available in a very limited number of health centers in the country, and molecular tests for dengue were not available in the country. As a consequence, plasma samples collected and stored at -20°C since 2014 were analyzed only in 2019. Although RDT for dengue was performed on the spot and yielded a positive result in all 27 samples obtained in Nouakchott in 2014, the lengthy delay before molecular analysis and transportation of samples which were dried on filter papers are probably two major reasons that explain why complete genome sequencing and envelope gene sequencing were unsuccessful in all 26 of 26 (100%) samples (real-time RT-PCR failed in 1 of 27 samples) and in 20 of 26 (76.9%) samples, respectively. These limitations do not put into question the positive results of RDT for dengue confirmed by real-time RT-PCR and the presence of DENV-2 in Nouakchott, at least in 2014, 2017, and 2018, as demonstrated in the present study. Other limitations include the unavailability of samples from later dengue epidemics that occurred in Nouakchott and elsewhere in the country, with the exception of two samples obtained through imported dengue cases in France. Moreover, a regular entomological surveillance has not been undertaken in Mauritanian cities where dengue epidemics occurred. These difficulties are compounded by the lack of national dengue control program, which should include implementation of measures to increase awareness about the disease in the general population through health education.

## Conclusions

Despite these limitations of the study, our findings suggest the emergence of an epidemic DENV-2 strain in Mauritania during the 2014 Nouakchott outbreak, most probably transmitted by *Ae*. *aegypti*. This DENV-2 strain has been found in other West African countries through travel-acquired infections, in particular in countries where DENV-2 had not been previously detected. Although our data seem to suggest a recent and rapid dispersion of DENV-2 throughout the region, other three DENV serotypes are also present in West Africa. The rapid propagation of DENV may be due, at least in part, to increased commercial exchanges and population movements in the region, dispersing both the vector and the virus to previously spared arid areas, such as in northern Mauritania. The possible relationship between urban DENV transmission and sylvatic cycle known to exist in the sub-region needs to be further investigated. More complete genome sequences of DENV-2 from West Africa could provide a better understanding of the dynamics of its circulation. There is also an urgent need for DENV and arboviral surveillance and outbreak forecasting in West Africa.
